# A Gene Signature to Determine Metastatic Behavior in Thymomas

**DOI:** 10.1371/journal.pone.0066047

**Published:** 2013-07-24

**Authors:** Yesim Gökmen-Polar, Robert W. Cook, Chirayu Pankaj Goswami, Jeff Wilkinson, Derek Maetzold, John F. Stone, Kristen M. Oelschlager, Ioan Tudor Vladislav, Kristen L. Shirar, Kenneth A. Kesler, Patrick J. Loehrer, Sunil Badve

**Affiliations:** 1 Department of Medicine, Indiana University School of Medicine, Indianapolis, Indiana, United States of America; 2 Department of Pathology and Laboratory Medicine, Indiana University School of Medicine, Indianapolis, Indiana, United States of America; 3 Center for Computational Biology and Bioinformatics, Indiana University School of Medicine, Indianapolis, Indiana, United States of America; 4 Department of Surgery, Indiana University School of Medicine, Indianapolis, Indiana, United States of America; 5 Castle Biosciences Incorporated, Friendswood, Texas, United States of America; 6 The DNA Diagnostics Laboratory, St. Joseph's Hospital and Medical Center, Phoenix, Arizona, United States of America; 7 Castle Biosciences Incorporated, Phoenix, Arizona, United States of America; The University of Hong Kong, China

## Abstract

**Purpose:**

Thymoma represents one of the rarest of all malignancies. Stage and completeness of resection have been used to ascertain postoperative therapeutic strategies albeit with limited prognostic accuracy. A molecular classifier would be useful to improve the assessment of metastatic behaviour and optimize patient management.

**Methods:**

qRT-PCR assay for 23 genes (19 test and four reference genes) was performed on multi-institutional archival primary thymomas (*n* = 36). Gene expression levels were used to compute a signature, classifying tumors into classes 1 and 2, corresponding to low or high likelihood for metastases. The signature was validated in an independent multi-institutional cohort of patients (*n* = 75).

**Results:**

A nine-gene signature that can predict metastatic behavior of thymomas was developed and validated. Using radial basis machine modeling in the training set, 5-year and 10-year metastasis-free survival rates were 77% and 26% for predicted low (class 1) and high (class 2) risk of metastasis (*P* = 0.0047, log-rank), respectively. For the validation set, 5-year metastasis-free survival rates were 97% and 30% for predicted low- and high-risk patients (*P* = 0.0004, log-rank), respectively. The 5-year metastasis-free survival rates for the validation set were 49% and 41% for Masaoka stages I/II and III/IV (*P* = 0.0537, log-rank), respectively. In univariate and multivariate Cox models evaluating common prognostic factors for thymoma metastasis, the nine-gene signature was the only independent indicator of metastases (*P* = 0.036).

**Conclusion:**

A nine-gene signature was established and validated which predicts the likelihood of metastasis more accurately than traditional staging. This further underscores the biologic determinants of the clinical course of thymoma and may improve patient management.

## Introduction

Thymomas and thymic carcinomas are rare epithelial tumors arising from the thymus gland in the anterior mediastinum. The exact incidence of thymomas is not well documented, but estimated at 0·15/100,000 person-years [1]. The rarity and morphological heterogeneity of these tumors have significantly contributed to difficulties in predicting the behaviour of these tumors.

The World Health Organization (WHO) unifying schema recognizes “organotypic” (thymus-like) type A, AB, B1, B2, and B3 thymomas and “nonorganotypic” thymic carcinomas. The types A, AB, and B1 were believed to be benign with rare recurrences/progression of disease [2], [3], while B3 tumors were aggressive. However, the WHO schema is difficult to apply uniformly; in addition, a significant number of tumors show mixed morphological features [4]. The Masaoka system is most commonly used to stage thymomas. Briefly, stages I and II represent tumors that are confined to the thymus, the latter with limited local invasion. Stage III tumors demonstrate involvement of adjacent structures and stage IV includes transpleural and hematogenous spread. Recent studies have documented recurrences and metastasis in all types of thymomas, irrespective of subtype or resection status, highlighting the limitations of current assessment strategies [5]–[7]. As a result, better tools are needed to predict the metastatic behavior of thymomas more accurately. Our prior gene expression microarray analysis identified a number of genes that could predict metastatic behavior in thymomas [8]. Of note, these genes were not part of the proliferation metagene but had functions related to invasion [9], [10]. In this study, we report the development and validation of a nine-gene qRT-PCR assay that predicts the likelihood of metastasis using a relatively large multi-institutional series of thymomas. This assay categorizes patients into low (class 1) and high (class 2) risk and enables a novel biologic-based clinical approach for decision making in patients with this rare malignancy.

## Methods

### Patients

This multicenter cohort study consists of patients surgically treated at multiple institutions prior to being referred for second opinion to The Indiana University Simon Cancer Center (IUSCC). We analyzed 111 cases of thymomas with followup and archival blocks requested from multiple institutions (Figure S1). The histology was reviewed by a single pathologist (SB) using the WHO criteria; cases with multiple histological subtypes were categorized on the basis of the predominant subtype. In accordance with prior studies, low risk of metastasis tumors (types A, AB, and B1) were grouped together for the purposes of analysis [2], [3]. Thirty-six samples were selected for the training set based upon shortest and longest time to metastasis. The remaining samples were included in the validation set. Clinical data on all patients were obtained by chart review or from the referring physician. Studies were approved by the Institutional Review Board of Indiana University.

### Sample preparation

The blocks were entirely composed of tumor in all but three cases; in these three cases macrodissection was performed. RNA was extracted from five 10-µm-thick sections in a College of American Pathologists (CAP)-accredited, Clinical Laboratory Improvement Amendments (CLIA)-certified laboratory (see Appendix S1).

### Assay methods and calculation of low and high metastatic potential of gene expression signature

Prediction Analysis of Microarrays (PAM) identified ten genes associated with metastases and nine genes associated with stage (Figures S2A and S2B; Tables S2 and S3) [11]. qRT-PCR analysis for these 19 discriminant genes (both metastases and stage related) and four control genes (Table S1) from formalin-fixed, paraffin-embedded (FFPE) primary thymomas was performed under standard operating procedures at the DNA Diagnostics Laboratory at St. Joseph's Hospital and Medical Center under a work-for-hire agreement with Castle Biosciences (details in Appendix S1). Delta threshold cycle (ΔC_t_) values for each of the 19 genes of interest were calculated by subtracting from the average C_t_ for each target gene the GeoMean of the C_t_ values from the four reference genes. ΔC_t_ values were standardized according to the mean of the expression of target genes in the training set, with a scale equivalent to the standard deviation. Nonlinear radial basis machine (RBM) classification was based upon the normalized ΔC_t_ value for each gene of the combined 19-gene signature. RBM utilizes the GLIMMIX procedure in SAS to fit a radial smoothing kernel according to the continuous variable of gene expression. Similar to support vector machine, RBM transforms the gene measurements using a kernel function in order to find an optimal hyperplane in multivariate dimension, thus providing a predicted classification able to separate the 36-sample training set into classes 1 and 2, corresponding to low or high risks. The nine-gene signature determined for subsequent validation set tissue was compared to the RBM-determined genetic profile of the training set.

### Study endpoints

The primary endpoint was to determine whether the gene signature could accurately predict 5- and 10-year metastasis-free survival (MFS), defined as time from diagnosis to the development of multifocal pleural/lung deposits or extrathoracic metastases. One of the primary reasons for choosing this endpoint is that evaluation of the mediastinum for recurrence, following surgery and radiation therapy, is difficult. The secondary endpoint was to perform comparative analyses with Masaoka staging system, completeness of resection, and the WHO histological type to determine whether it was an independent predictor.

### Statistical analysis

Using a training set, multiple nonlinear predictive modeling methods were performed to assess the prognostic ability of the gene signature to identify the best classifier. In addition to RBM, partition tree analysis, K-nearest neighbor analysis, and distance scoring analysis were performed using the SAS-based JMP Genomics software. The area under the receiver operator characteristic curve (ROC) was calculated for each analysis to assess the predictive probabilities of each method. Survival analysis was performed using Kaplan–Meier plots and log-rank analysis. Cox regression analysis was performed using WinSTAT software for the variables age, gender, stage (I/II vs. III/IV), WHO type, completeness of resection, autoimmune disease, and gene signature. Impact of chemotherapy was also analyzed. Using WinSTAT, 95% confidence interval (95% CI) ranges for hazard ratios were calculated.

## Results

### Characteristics of the patients

Demographic and clinical characteristics of the patients and followup information were acquired from medical charts and referring physicians (Table 1). All patients with stage IV tumors received chemotherapy. Twelve of 43 nonmetastatic patients in the validation set received multimodality treatment. In the nonmetastatic patients, chemotherapy was not significant in predicting MFS (data not shown). Selection of training set samples was primarily based on shortest time to metastasis (average time to metastasis, <1 yr) and, for nonmetastatic cases, longest followup (>5 yrs; median followup, 8.4 yrs.).

**Table 1:** Baseline demographics of training set and independent validation set.

	Training		Validation	
	Metastatic	Non-Metastatic	Metastatic	Nonmetastatic
	(*n* = 21)	(*n* = 15)	(*n* = 32)	(*n* = 43)
Median MFS/Followup (range)	0.86 (0–13.0)	8.41 (5.1–18.1)	2.35 (0–11.2)	1.87 (0.1–13.8)
Median Age (range)	46 (34–85)	48 (31–62)	43 (27–68)	57 (18–79)
Gender				
Male	6 (29%)	5 (33%)	17 (53%)	16 (37%)
Female	15 (71%)	10 (67%)	15 (47%)	27 (63%)
Masaoka Stage				
I	2 (10%)	10 (67%)	6 (19%)	16 (37%)
II	3 (14%)	5 (33%)	3 (9%)	13 (28%)
III	5 (24%)	0 (0%)	13 (41%)	14 (33%)
IV	11 (52%)	0 (0%)	10 (31%)	0 (0%)
WHO Classification				
A	1 (5%)	0 (0%)	0 (0%)	7 (16%)
AB	3 (14%)	5 (33%)	3 (9%)	15 (35%)
B1	1 (5%)	4 (27%)	10 (31%)	7 (16%)
B2	8 (38%)	3 (20%)	14 (44%)	12 (28%)
B3	8 (38%)	3 (20%)	4 (13%)	2 (5%)
Extent of Resection				
No evidence of disease	9 (43%)	14 (93%)	14 (44%)	37 (86%)
Residual disease	12 (57%)	1 (7%)	18 (56%)	6 (14%)
Autoimmune Disease				
Yes	10 (48%)	7 (47%)	11 (34%)	12 (28%)
No/not stated	11 (52%)	7 (47%)	21 (66%)	31 (73%)
Adjuvant CT and/or RT				
Surgery alone	3 (14%)	12 (80%)	6 (19%)	29 (67%)
Chemotherapy	2 (10%)	0 (0%)	1 (3%)	1 (2%)
Surgery/CT	8 (38%)	1 (7%)	9 (28%)	6 (14%)
Surgery/RT	4 (19%)	3 (20%)	6 (19%)	4 (9%)
Trimodal therapy	3 (14%)	0 (0%)	9 (28%)	2 (5%)

*CT, chemotherapy; MFS, metastasis-free survival; RT, radiation therapy*.

### Prognostic impact of nine-gene signature in relation to stage, WHO histological type, and residual disease

**Figure 1:**
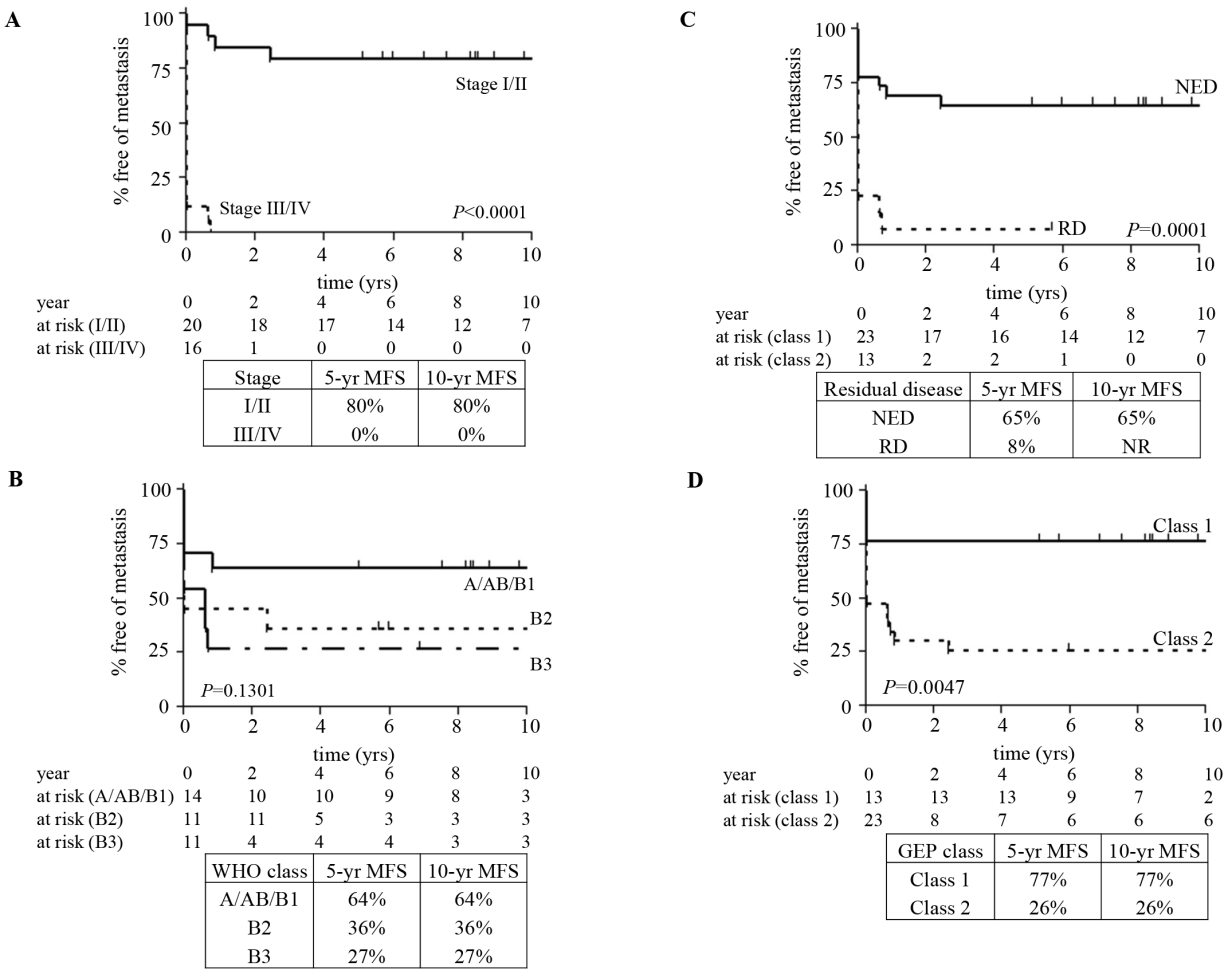
Kaplan-Meier curves for metastasis-free survival (MFS) in the training set cohort of samples. MFS is grouped according to Masaoka staging (A), WHO classification (B), extent of resection (C), or predicted nine-gene signature class 1 (low metastatic potential) or class 2 (high metastatic potential) as determined by radial basis machine predictive modeling algorithm (D). Numbers of cases at risk at two-year time points are shown below each graph, and 5- and 10-year MFS is presented. *P* values for each classification system were calculated by log-rank method. *GEP, gene expression profile; NED, no evidence of disease; RD, residual disease, WHO, World Health Organization*.

**Figure 2:**
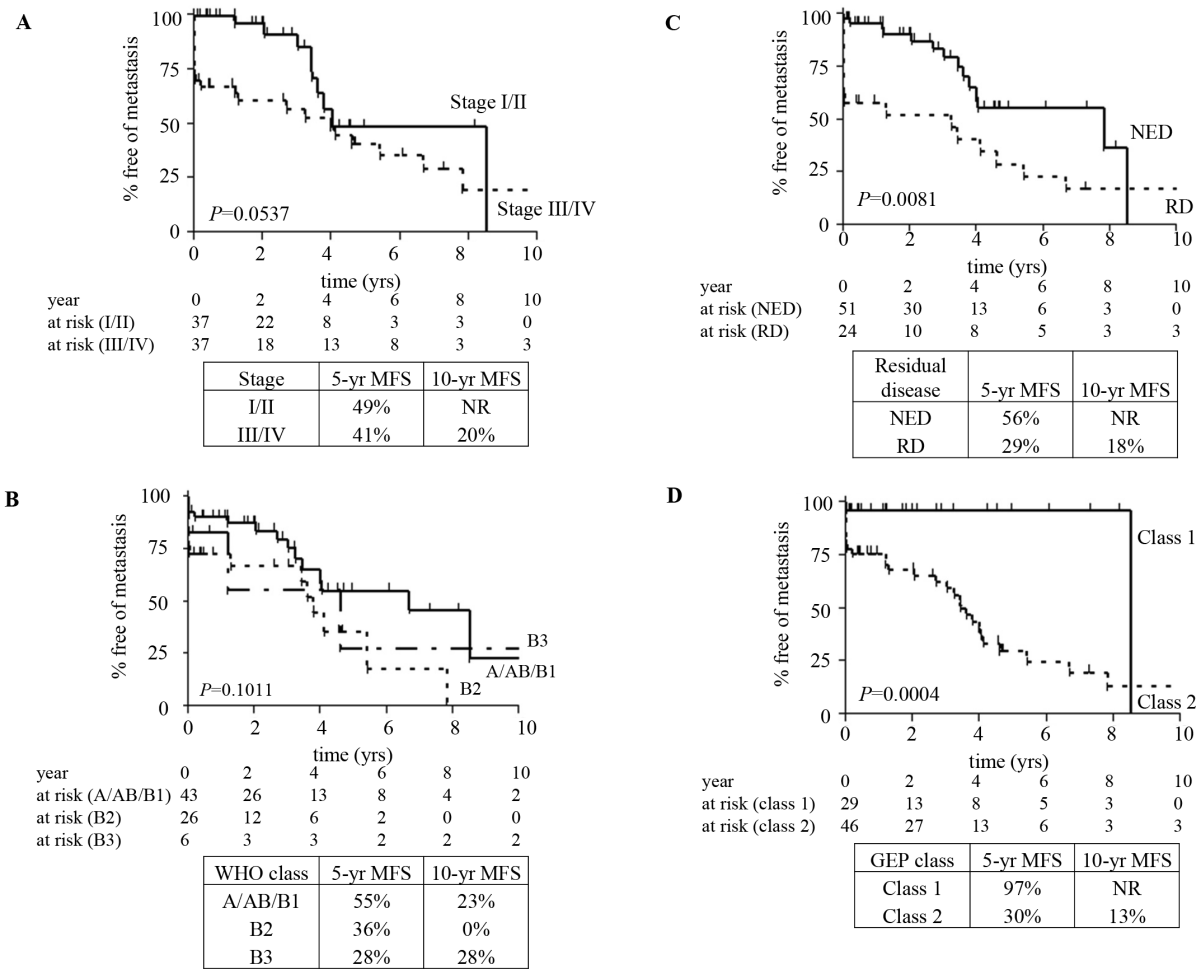
Kaplan-Meier curves for metastasis-free survival (MFS) in the validation set cohort of samples. MFS is grouped according to Masaoka staging (A), WHO classification (B), extent of resection (C), or predicted nine-gene signature class 1 (low metastatic potential) or class 2 (high metastatic potential) as determined by radial basis machine predictive modeling algorithm (D). Numbers of cases at risk at two-year time points are shown below each graph, and 5- and 10-year MFS is presented. *P* values for each classification system were calculated by log-rank method. *GEP, gene expression profile; NED, no evidence of disease; RD, residual disease, WHO, World Health Organization*.

A number of models were analyzed in the training set; this resulted in identification of two models; a nine-gene signature (Table 2) and a 19-gene signature (Table S1), both of which showed similar performance characteristics in the validations set (see Figures 1 and 2; Figure S3). The nine-gene signature was used in further analysis as it used only genes identified as metastases associated in the PAM analysis.

**Table 2:** The 9- gene panel to determine the metastatic behavior of thymomas.

Genes with increased expression in tumors with metastatic behavior	Genes with decreased expression in tumors with metastatic behavior	Reference genes
***AKR1B10***	***DACT3***	*IPO8*
***JPH1***	***SLC9A2***	*TFRC*
***NGB***	***PDGFRL***	*UBC*
	***FCGBP***	*PGK1*
	***PRRX1***	
	***SERPINF1***	

In the training set (*N* = 36), 10-year MFS according to Masaoka stage was 80% for patients with locally confined disease (stage I/II; *n* = 20) and 0% for extensive disease (stage III/IV; *n* = 16) (Figure 1A). WHO classification analysis was condensed to three groups: tumors that predominantly follow a benign course (types A, AB, and B1; *n* = 14), tumors that are thought to be low-grade malignant (type B2; *n* = 11), and tumors that can have an aggressive course (type B3; *n* = 11). The training set 10-year MFS rates were 64%, 36%, and 27% for groups A/AB/B1, B2, and B3, respectively (Figure 1B). The 10-year MFS rates for cases with no evidence of disease was 65% (n = 23), compared to an 8% rate in cases with residual disease (*n* = 13; Figure 1C). The nine-gene signature exhibited 10-year MFS rates of 77% for predicted low-risk and 26% for high-risk samples (Figure 1D).

In the independent validation set (*N* = 75), the 5-year MFS rate was 49% for stage I/II samples; there were no additional events at 10 years. The 5- and 10-year MFS rates were 41% and 20% for stage III/IV samples, respectively (Figure 2A). For the three WHO groups analyzed, 5- and 10-year MFS rates were 55%, 36%, and 28% and 23%, 0%, and 28% for groups A/AB/B1, B2, and B3, respectively (Figure 2B). Validation cases with no evidence of disease had a 5-year metastasis rate of 56%, while the 10-year time point was not reached. Cases exhibiting residual disease had 5- and 10-year rates of 29% and 18%, respectively (Figure 2C). Only completeness of resection achieved statistical significance (*P* = 0.0081). The nine-gene signature identified 29 patients as low-risk and 46 patients as high-risk. Kaplan-Meier analysis revealed a highly significant difference in MFS between the two classes (*P* = 0.0004). Five-year survival was 97% for low-risk with no additional events at 10 years (Figure 2D). Conversely, 5- and 10-year MFS for high-risk samples was 30% and 13%, respectively.

The nine-gene signature could reliably predict development of metastases in both the training (ROC, 0.85) and validation sets (ROC, 0.86). The metastasis prediction models based on Masaoka stage had ROC values of 0.88 and 0.69 for the training and validation cohorts, respectively, while models based on extent of resection had ROC values of 0.75 and 0.71, respectively. The nine-gene signature was significantly more accurate in classifying the patients into low- or high-risk metastasis group (*P* = 0.0001; sensitivity, 94%).

Positive (PPV) and negative (NPV) predictive values were calculated for gene signature, staging system, and extent of resection to show the precision of each method for predicting which tumors are at low and high risk of metastasis. The NPV showed that the gene signature was more precise than staging and absence of residual disease (93% vs. 76% and 73%, respectively) for identifying low-risk patients. PPV was comparable between gene signature stage and presence of residual disease (65%, 61%, and 75%, respectively). The specificity could have been compromised by two parameters, 1) short followup and 2) surgical and adjuvant therapies. Of the 16 patients classified as class 2 without metastases, only two patients had followup greater than 5 years. In addition, nine of these patients had no evidence of disease following treatment for the thymoma and nine had received adjuvant therapies.

### Accuracy of nine-gene signature is independent of the modeling method

Various predictive modeling methods including Partition-tree analysis, K-nearest neighbor, and distance scoring, were used to predict metastatic risk in the validation sample cohort. The results shown in Table 2 demonstrate that the accuracy and sensitivity is not significantly affected by the predictive algorithm used. Further, Kaplan–Meier curves generated from the predicted outcomes determined by each model show highly statistically significant long-term MFS rates between low- and high-risk classes.

### Heterogeneity analysis

The impact of tumor heterogeneity on consistency of the signature showed discordance in only 1/24 (4%) tumor blocks (for details see Table S4). The single discordant case was from a tumor with no evidence of metastasis, but classified as high risk by nine-gene signature. Three other blocks from the same tumor were classified as low risk. Importantly, all cases (11/11 regions) with known metastases were predicted to be high-risk tumors. These results support the conclusion that intratumoral heterogeneity does not significantly affect the accuracy of the signatures.

### Univariate and multivariate Cox regression analysis of clinical factors in relation to the prediction of metastasis

In univariate Cox regression analysis, the nine-gene signature was an accurate classifier of metastases (hazard ratio [HR] = 8.33; 95% CI = 1.99–34.9; *P* = 0.004; Table 3). The presence of residual disease and age were also significant factors for classifying metastasis (HR = 2.51; 95% CI = 1.22–5.15; *P* = 0.012 and HR = 0.43; 95% CI = 0.2–0.91; and *P* = 0.028, respectively). Masaoka stage I/II vs. stage III/IV approached statistical significance having a 2.09 HR (95% CI = 0.95–4.59; *P* = 0.067; Table 4). Distribution of gene signature class by stage and completeness of resection is shown in Table S5. WHO schema, gender, and autoimmune disease were not significantly associated with the likelihood of metastasis. Adjuvant chemotherapy and/or radiation therapy were also not significant (data not shown).

**Table 3:** Accuracy of nonlinear modeling algorithms for predicting metastatic risk in a 75-sample thymoma validation set.

Predictive Model	ROC	Sensitivity	Kaplan–Meier *P* value (log-rank)
Radial basis machine	0.86	0.94	0.0004
K-nearest neighbor	0.85	0.94	0.0011
Partition tree	0.85	0.91	0.0019
Distance scoring	0.84	0.88	0.0029

*ROC, receiver operator characteristic curve*.

**Table 4:** Univariate and multivariate analysis of the independent 75-sample validation set.

	Cox univariate analysis		Cox multivariate analysis		Kaplan-Meier
	HR (*P* value*)	95% CI	HR (*P* value)	95% CI	*P* value
Age >50 years	0.43 (0.028)	0.20–0.91	0.50 (0.128)	0.21–1.22	0.0207
Gender	1.18 (0.648)	0.58–2.38	–	–	–
Autoimmune disease	1.42 (0.353)	0.68–2.99	–	–	–
Residual disease	2.51 (0.012)	1.22–5.15	1.76 (0.214)	0.72–4.31	0.0081
Stage III/IV	2.09 (0.067)	0.95–4.59	–	–	–
WHO class	1.46 (0.116)	0.91–2.33	–	–	–
GEP Class 2 (high risk)	8.33 (0.004)	1.99–34.9	5.26 (0.036)	1.12–24.8	0.0004

*CI, confidence interval; HR, hazard ratio*.

* ***P≤0.05 is considered as statistically significant***.

In multivariate Cox analysis, only the nine-gene signature was an independent predictor of metastasis (HR = 5.26; 95% CI = 1.12–24.8; *P* = 0.036). The result illustrates the utility of the gene signature in combination with other factors commonly used today.

## Discussion

Postoperative therapeutic management has predominantly been subjective and loosely based upon Masaoka stage, presence of residual disease, and, to a limited extent, WHO type [12]–[14]. Recent studies have shown that all types of thymic tumors, regardless of histologic type or completeness of resection, can be associated with invasion, thoracic metastases, and extrathoracic metastases [5]–[7]. Without level one evidence, the National Comprehensive Cancer Network guidelines propose adjuvant radiation or chemoradiation therapy for all patients with resected stage II/III tumors, irrespective of other factors mentioned above [15]. This leads to undertreatment of patients with aggressive tumors diagnosed at an early stage of disease as well as overtreatment of indolent tumors diagnosed at a later stage. A more accurate method to identify metastatic behavior could result in prescription of adjuvant therapies to these likely patients. Patients at low or no risk can be spared from the toxicities associated with chemo- and radiation therapies. In addition, the presence of biologically heterogeneous cases in clinical trials could result in biased conclusions regarding therapeutic efficacy of drugs.

A number of groups have tried to develop prognostic/predictive markers for thymomas. Most of these studies have correlated histologic type and tumor stage with markers of proliferation, cell death, migration, or invasion [16]–[20]. Molecular profiling technologies have identified differentially regulated genes between different histologic types [21]–[23]. However, histology-based hierarchical clustering of gene expression data did not show an association with relapse or metastases in our prior study [8].

In this study, we have developed and validated a nine-gene signature and compared its prognostic value with other clinicopathological parameters. In univariate analysis, apart from the gene signature, age (*P* = 0.028) and completeness of excision were significant (*P* = 0.012) and Masaoka stage approached significance (*P* = 0.067). Of note, only 1/29 patients classified as class 1 by gene signature developed metastases. In contrast, 16/46 patients classified as class 2 developed no evidence of metastases to date. Upon closer examination of these 16 cases, nine had complete excision of the tumor and the remainder received adjuvant therapy. More importantly, only two of the 16 patients had followup data for more than 5 years. These parameters could have resulted in the improved observed outcomes in these patients. Thus, the ability of the assay to accurately identify metastatic cases is likely to be higher than reflected in the current study. Furthermore, we confirmed that the signature was not significantly affected by intratumoral heterogeneity. The nine-gene signature is the first prognostic molecular signature that can accurately predict metastatic behavior in thymomas. It was particularly efficient at predicting which patients would not develop metastases; further studies need to be carried out to analyze whether these patients can be spared from adjuvant chemoradiation therapy.

Among the nine genes included in the signature, three genes (*AKR1B10*, *JPH1*, and *NGB*) were upregulated in class 2 patients. These genes have been previously associated with invasion and metastasis or chemoresistance in multiple cancers [24]–[29], where they have been considered as potential therapeutic candidates. The signature is not dependent on proliferation as the genes do not belong to a proliferation metagene [9], [10]. The expression levels of *AKR1B10* are low (unpublished data) in IU-TAB-1 cell line, established by our group from a patient with stage II thymoma, WHO type AB [30]. Further mechanistic studies, including knock-in and knock-out approaches, are being undertaken to assess the therapeutic potential of these genes. The nine-gene signature not only could improve prognostication for all thymoma patients, but could also identify potential druggable targets for patients with high metastatic potential.

The current retrospective study was based on multi-institutional samples from patients receiving different surgical and postsurgical treatments. Given the rarity of this disease, the initial surgical management, the assessment of extent of disease, and, in some cases, postoperative therapy (chemotherapy and/or radiation therapy) was often provided at local centers prior to referral to the IUSCC. This can be considered a strength of the study as it permits generalization of the results to patients with thymomas and enables personalized management based on their risk. To date, no prospective surgical trial has been performed on thymoma, but this assay potentially allows stratification of patients with locally advanced disease into low- (surveillance) or high- (interventional group) risk categories with treatments assigned appropriately, which is planned to be evaluated prospectively by ECOG-ACRIN.

The signature has the potential to change clinical practice in a number of ways. In the preoperative setting, a high score obtained from a biopsy specimen might alert the surgeon about the possibility of a more extensive disease and perhaps the need for assistance from an expert in the field. Postoperatively, a number of adjuvant chemotherapy and radiation therapy regimens have been used in an empirical manner. A regimen may be falsely thought to be effective if used on patients whose tumors have low metastatic potential. The ability to stratify patients into risk groups will permit proper assessment of therapeutic impact. Currently, the rationale for selecting and trying out therapeutic regimens for thymomas is the efficacy of these treatments for other cancers. The identification of nonproliferation-associated druggable target genes may enable selection and trials of appropriate therapies, paving a path towards personalized medicine.

In conclusion, we have developed and validated a nine-gene prognostic assay that serves as an independent predictor of MFS and appears superior to currently-used prognosticators such as Masaoka stage and histology. The current study provides a useful template for the efficient application of genetic expression data for the patient's benefit, especially in rare diseases.

## Supporting Information

Appendix S1 **This appendix has been provided by the authors to give readers additional information about their work.**
(DOCX)

Figure S1 **Consort diagram showing cases of thymomas used in the gene expression analysis.**
(DOCX)

Figure S2 **A.** Prediction of lack of metastasis using PAM. **B.** Prediction of the presence of stage I/II using PAM.
(DOCX)

Figure S3 **Kaplan-Meier curve of metastasis-free survival (MFS) determined using the 19-gene predictor.**
(DOCX)

Table S1 **24-gene panel used to develop prognostic signature for metastatic behavior in thymomas.** Bold genes are those included in the final nine-gene predictor. Of note, these nine genes were all included in the panel of ten genes identified as associated with metastases in the PAM signature developed using microarrays.
(DOCX)

Table S2 Cross-validation confusion matrix for predicting lack of metastasis*.
(DOCX)

Table S3 Cross-validation confusion matrix for predicting early stage*.
(DOCX)

Table S4 **Heterogeneity analysis in eight cases of thymoma.**
(DOCX)

Table S5 **Distribution of gene signature class by stage and completeness of resection.**
(DOCX)
