# Supplementary material for: A Gene Signature to Determine Metastatic Behavior in Thymomas
Source: PLoS One. 2013 Jul 24;8(7):e66047. doi: 10.1371/journal.pone.0066047 (PMC3722217; doi:10.1371/journal.pone.0066047)
Supplement: Appendix S1 — This appendix has been provided by the authors to give readers additional information about their work. (DOCX) [file pone.0066047.s001.docx]

**Supplementary Appendix**

This appendix has been provided by the authors to give readers additional information about their work.

Supplement to: Gökmen-Polar Y, Cook RW, Goswami CP, et al. A Gene Signature to Determine Metastatic Behavior in Thymomas

**Supplemental Methods**

***Section I. Sample Preparation and Selection***

The nine-gene signature was developed in a training set composed of archival formalin-fixed, paraffin-embedded (FFPE) tumor blocks from patients with thymomas. All steps of the assay, including the prognostic algorithm, were fully developed, completely specified, and technically validated in line with CLIA-laboratory guidelines before initiation of the independent validation. Laboratory personnel were blinded to all clinical outcomes data.

*Sample Selection*

Of the over 600 cases of thymic tumors seen at IUSCC, 76 cases of thymoma with archival blocks were available for the study (Figure S1). In additional 49 cases, blocks could be obtained from the primary institution to perform gene expression analyses. Of these 125 cases, 111 samples met both pathological and Clinical Laboratory Improvement Amendments (CLIA) quality control standards. Fourteen samples did not meet CLIA approved standards.

***Section II. Selection of 19 Target Genes for qRT-PCR Using Prediction Analysis of Microarrays***

Supervised class prediction analyses were performed by using Prediction Analysis of Microarrays (PAM) software. This program is based on the method of the nearest shrunken centroids to determine a subgroup of genes that best represents a predefined class (Table S1). PAM identified a gene set composed of ten genes that predicted the lack of metastases with a 4% error rate. Similar analysis for stage showed that a set of nine genes predicted the presence of early-stage (stage I or II) disease with a 9% error rate. Individual cross-validation plots were shown for (Figure S2A) Metastasis Yes phenotype (Yes, blue) and Metastasis No phenotype (No, blue). The threshold value (1.56) was chosen to use for shrinkage and prediction with a 4% error rate. Individual cross-validation plots were shown for stage I/II (green) and stage III/IV (blue) disease (Figure S2B; Table S2). The threshold value (1.73) was chosen to use for shrinkage and prediction with a 9% error rate (Table S3).

Reference genes were determined using Human Endogenous Control Array (Applied Biosystems) and GeNorm® software. The gene signature was selected based on the smallest prediction error combining both groups representing metastatic behavior and stage. Expression of the 19 genes identified in the PAM analysis of microarray expression data was analyzed by RT-PCR in all 111 thymoma tumors. Multiple prognostic models were analyzed in the training set. A gene signature could be developed using nine genes (see main manuscript) as well as all the 19 genes; both the signatures were significant in the validation set. In brief, the 19-gene signature identified 30 patients at low risk and 45 patients at high risk of metastasis. Kaplan-Meier analysis shows a significant difference between the two classes (*P*=0.0075), with a 5-year MFS of 76% for low-risk patients and 35% for high risk patients (Figure S3). However, a nine-gene signature developed from only the metastases related genes (from PAM) showed better performance characteristics and was selected for further analyses.

***Section III. Assay Methods***

**Assay Methods**

*Tissue processing and RNA isolation*

Five 10-µm-thick FFPE sections obtained from primary thymoma tumors were macrodissected and collected into a single 1.5ml microcentrifuge tube. Tissue was deparaffinized and processed according to the AmbionRecoverAll Total Nucleic Acid Isolation Kit (Life Technologies, Grand Island, NY) protocol. Following nucleic acid isolation, protein degradation, and RNA isolation procedures as described in RecoverAll Kit, RNA was eluted from the filter cartridge with 60µl of nuclease-free water.

*cDNA Generation and RT-PCR Analysis*

All nucleic acid quantitation and evaluation steps were performed using the Nanodrop 1000 spectrophotometer according to the manufacturer’s instructions. Conversion of 1µg of RNA to cDNA was carried out in a 20µl reaction using the Applied Biosystems High Capacity cDNA Reverse Transcription Kit (Life Technologies). Following cDNA synthesis, a 14-cycle preamplification step was performed in a 96-well plate for all samples using pooled TaqMan assays from the 23 genes (19 discriminant and four reference genes). Preamplification reactions were removed to 1·5ml centrifuge tubes and diluted 1:20 (final volume = 1ml) with 1X TE buffer.

TaqMan reactions were performed in duplicate using custom array microfluidic cards preloaded with TaqMan Gene Expression Assays containing the 23 genes and the manufacturer’s instructions were followed. Briefly, 50µl of the preamplified sample was diluted 1:2 using Applied Biosystems TaqMan 2X Gene Expression Master Mix and loaded into the fill port of the custom array cards. RT-PCR was carried out in an Applied Biosystems 7900HT Fast Real-Time PCR System.

***Section IV. Heterogeneity Analysis***

Eight thymoma cases were assessed for heterogeneity between tumor blocks using the RT-PCR expression analysis described previously for training and validation set samples. As shown in Table S4, heterogeneity has low impact upon the consistent assignment of risk to multiple regions of the same tumor. Using the nine-gene predictor, only one region of 24 was discordantly classified (Table S4).

***Section V: Distribution of Gene Signature Class by Stage and Completeness of Resection***

See Table S5.
