# Supplementary figures and images for: A Gene Signature to Determine Metastatic Behavior in Thymomas
Source: PLoS One. 2013 Jul 24;8(7):e66047. doi: 10.1371/journal.pone.0066047 (PMC3722217; doi:10.1371/journal.pone.0066047)

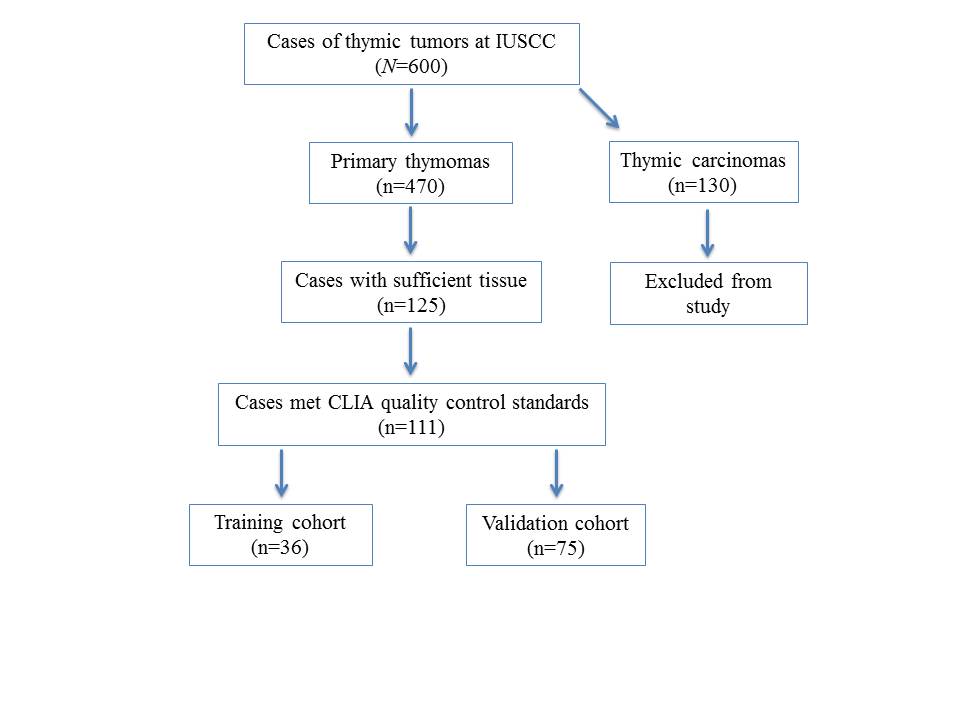

Supplement: Figure S1 — Consort diagram showing cases of thymomas used in the gene expression analysis. (DOCX) [file pone.0066047.s002.docx]
