# Supplementary material for: A Gene Signature to Determine Metastatic Behavior in Thymomas
Source: PLoS One. 2013 Jul 24;8(7):e66047. doi: 10.1371/journal.pone.0066047 (PMC3722217; doi:10.1371/journal.pone.0066047)
Supplement: Figure S3 — Kaplan-Meier curve of metastasis-free survival (MFS) determined using the 19-gene predictor. (DOCX) [file pone.0066047.s004.docx]

% free of metastasis

100

75

50

25

0

time (yrs)

0 2 4 6 8 10

low risk

high risk

*P*=0.0005

**A. Training set 19-genes**

year

at risk (low)

at risk (high)

0

13

23

2

13

8

4

13

7

6

9

6

8

7

6

10

2

6


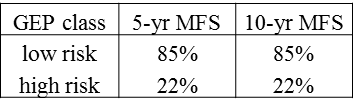


**B. Validation set 19-genes**


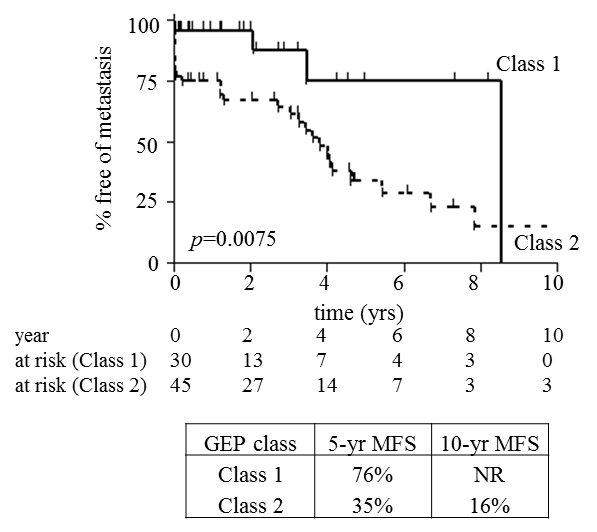


*GEP, gene expression profile*
