# Supplementary material for: A Gene Signature to Determine Metastatic Behavior in Thymomas
Source: PLoS One. 2013 Jul 24;8(7):e66047. doi: 10.1371/journal.pone.0066047 (PMC3722217; doi:10.1371/journal.pone.0066047)
Supplement: Table S1 — 24-gene panel used to develop prognostic signature for metastatic behavior in thymomas. Bold genes are those included in the final nine-gene predictor. Of note, these nine genes were all included in the panel of ten genes identified as associated with metastases in the PAM signature developed using microarrays. (DOCX) [file pone.0066047.s005.docx]

**Table S1.** 24-gene panel used to develop prognostic signature for metastatic behavior in thymomas. Bold genes are those included in the final nine-gene predictor. Of note, these nine genes were all included in the panel of ten genes identified as associated with metastases in the PAM signature developed using microarrays.

| **Genes with increased expression in tumors with metastatic potential** | **Genes with decreased expression in tumors with metastatic potential** | **Reference genes** |
| --- | --- | --- |
| ***AKR1B10*** | ***DACT3*** | *IPO8* |
| *STC1* | *COL11A1* | *TFRC* |
| *STC2* | ***SLC9A2*** | *UBC* |
| ***JPH1*** | ***PDGFRL*** | *PGK1* |
| ***NGB*** | ***FCGBP*** |  |
| *SLC7A11* | ***PRRX1*** |  |
|  | ***SERPINF1*** |  |
|  | *SCUBE2* |  |
|  | *MAB21L2* |  |
|  | *LEPR* |  |
|  | *LCA5* |  |
|  | *GPR98* |  |
|  | *RSPO3* |  |
