# Supplementary material for: A Gene Signature to Determine Metastatic Behavior in Thymomas
Source: PLoS One. 2013 Jul 24;8(7):e66047. doi: 10.1371/journal.pone.0066047 (PMC3722217; doi:10.1371/journal.pone.0066047)
Supplement: Table S2 — Cross-validation confusion matrix for predicting lack of metastasis*. (DOCX) [file pone.0066047.s006.docx]

**Table S2**. Cross-validation confusion matrix for predicting lack of metastasis*

| True/Predicted | NO | YES | Class Error Rate |
| --- | --- | --- | --- |
| NO | 25 | 1 | 0.04 |
| YES | 4 | 5 | 0.44 |

*Threshold=1.56
