# Supplementary material for: A Gene Signature to Determine Metastatic Behavior in Thymomas
Source: PLoS One. 2013 Jul 24;8(7):e66047. doi: 10.1371/journal.pone.0066047 (PMC3722217; doi:10.1371/journal.pone.0066047)
Supplement: Table S3 — Cross-validation confusion matrix for predicting early stage*. (DOCX) [file pone.0066047.s007.docx]

**Table S3.** Cross-validation confusion matrix for predicting early stage*

| True/Predicted | I/II | III/IV | Class Error Rate |
| --- | --- | --- | --- |
| I/II | 20 | 2 | 0.09 |
| III/IV | 4 | 7 | 0.36 |

*Threshold=1.73
