# Supplementary material for: A Gene Signature to Determine Metastatic Behavior in Thymomas
Source: PLoS One. 2013 Jul 24;8(7):e66047. doi: 10.1371/journal.pone.0066047 (PMC3722217; doi:10.1371/journal.pone.0066047)
Supplement: Table S4 — Heterogeneity analysis in eight cases of thymoma. (DOCX) [file pone.0066047.s008.docx]

**Table S4.** Heterogeneity analysis in eight cases of thymoma

| Thymoma case | GEP Class Assignment | Metastasis status |
| --- | --- | --- |
| Case A | Class 2 | Y |
| Case A | Class 2 | Y |
| Case A | Class 2 | Y |
| Case A | Class 2 | Y |
| Case A | Class 2 | Y |
| Case B | Class 2 | N |
| Case B | Class 1 | N |
| Case B | Class 1 | N |
| Case B | Class 1 | N |
| Case C | Class 2 | N |
| Case C | Class 2 | N |
| Case C | Class 2 | N |
| Case C | Class 2 | N |
| Case C | Class 2 | N |
| Case D | Class 2 | N |
| Case D | Class 2 | N |
| Case E | Class 2 | Y |
| Case E | Class 2 | Y |
| Case F | Class 2 | Y |
| Case F | Class 2 | Y |
| Case G | Class 2 | Y |
| Case G | Class 2 | Y |
| Case H | Class 1 | N |
| Case H | Class 1 | N |
