# Supplementary material for: A Gene Signature to Determine Metastatic Behavior in Thymomas
Source: PLoS One. 2013 Jul 24;8(7):e66047. doi: 10.1371/journal.pone.0066047 (PMC3722217; doi:10.1371/journal.pone.0066047)
Supplement: Table S5 — Distribution of gene signature class by stage and completeness of resection. (DOCX) [file pone.0066047.s009.docx]

**Table S5.** Distribution of gene signature class by stage and completeness of resection

|  | # cases | # cases with RD |
| --- | --- | --- |
| Stage I nonmets called class 1 | 11 | 0 |
| Stage I nonmets called class 2 | 5 | 0 |
| Stage I mets called class 1 | 1 | 0 |
| Stage I mets called class 2 | 6 | 0 |
|  |  |  |
| Stage II nonmets called class 1 | 8 | 1 |
| Stage II nonmets called class 2 | 4 | 1 |
| Stage II mets called class 1 | 0 | n/a |
| Stage II mets called class 2 | 2 | 1 |
|  |  |  |
| Stage III nonmets called class 1 | 8 | 1 |
| Stage III nonmets called class 2 | 6 | 2 |
| Stage III mets called class 1 | 0 | n/a |
| Stage III mets called class 2 | 13 | 8 |
