# Supplementary material for: A Gene Signature to Determine Metastatic Behavior in Thymomas
Source: PLoS One. 2013 Jul 24;8(7):e66047. doi: 10.1371/journal.pone.0066047 (PMC3722217; doi:10.1371/journal.pone.0066047)
Supplement: Supplementary file 9 [file pone.0066047.s003.docx]

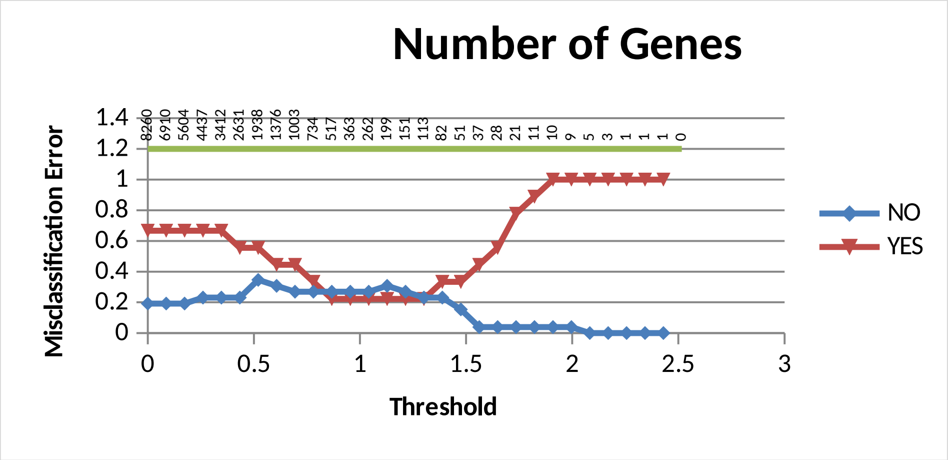
Number of Genes
8260 6910 5604 4437 3412 2631 1938 1376 1003 734 517 363 262 199 151 113 82 51 37 28 21 11 10 9 5 3 1 1 1 1 0
Misclassification Error
1.4
1.2
1
0.8
0.6
0.4
0.2
0
0 0.5 1 1.5 2 2.5 3
Threshold
NO
YES

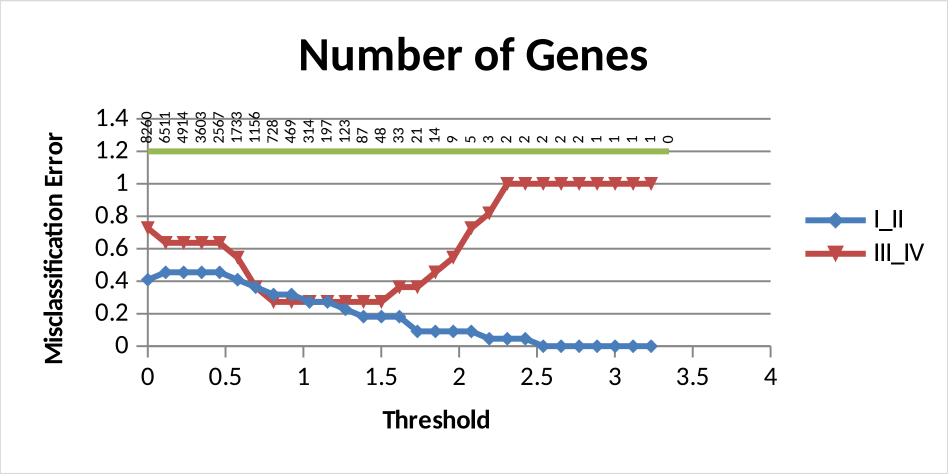
Number of Genes
8260 6511 4914 3603 2567 1733 1156 728 469 314 197 123 87 48 33 21 14 9 5 3 2 2 2 2 2 1 1 1 1 0
Misclassification Error
1.4
1.2
1
0.8
0.6
0.4
0.2
0
0 0.5 1 1.5 2 2.5 3 3.5 4
Threshold
I_II
III_IV
